# A Modified Single-Armed Suture Technique for Traumatic Cyclodialysis Cleft with Vitreoretinal Injury

**DOI:** 10.3390/jcm12134252

**Published:** 2023-06-25

**Authors:** Xueyong Zhang, Nan Wang, Guoli Zheng, Die Liu, Quyan Zhang, Wenbo Lei, Xiaobo Xia, Siqi Xiong

**Affiliations:** 1 Eye Center of Xiangya Hospital, Central South University, Changsha 410008, China; 2 Hunan Key Laboratory of Ophthalmology, Central South University, Changsha 410008, China; 3 National Clinical Research Center for Geriatric Disorders, Xiangya Hospital, Central South University, Changsha 410008, China

**Keywords:** cyclodialysis cleft, ocular trauma, vitreoretinal surgery, internal cyclopexy, ultrasound biomicroscopy

## Abstract

Our aim was to assess the therapeutic efficacy of a modified single-arm suture technique on traumatic cyclodialysis cleft with vitreoretinal injury. The procedure involved fixing a detached ciliary body using a single-armed 10-0 polypropylene suture under the assistance of a 29-gauge needle. Patients with a traumatic cyclodialysis cleft combined with an anterior and posterior segment injury who underwent modified internal cyclopexy together with vitreoretinal surgery were enrolled in this study. Ultrasound biomicroscopy (UBM) was used to diagnose and evaluate the cyclodialysis and anterior segment injury. B-scan ultrasonography was performed to assess the condition of the vitreous, retina and choroid. The surgical time and successful rate for repairing the cyclodialysis cleft were recorded. Preoperative and postoperative best-corrected visual acuity (BCVA), and intraocular pressure (IOP) were documented for assessment. The study included 20 eyes. The extent of the cyclodialysis cleft was from 30° to 360°. Besides a traumatic cyclodialysis cleft, the included cases also combined this with vitreous hemorrhages, retinal detachment, macular holes, choroid avulsion, and suprachoroidal hemorrhage. All the clefts were anatomically closed in one surgery. The average surgical time for fixing the cyclodialysis cleft was 2.68 ± 0.54 min/30° cleft. A significant improvement in LogMAR BCVA was observed from 2.94 ± 0.93 preoperatively to 1.81 ± 1.11 at the 6-month follow-up. IOP was elevated from 10.90 ± 6.18 mmHg preoperatively to 14.45 ± 2.35 mmHg at the 6-month follow-up. The modified single-armed suture technique was proved to be an effective method to fix the traumatic cyclodialysis cleft, which could facilitate the use of the procedure to repair chorioretinal disorders. It improved the BCVA and maintained the IOP with less postoperative complications.

## 1. Introduction

Cyclodialysis cleft refers to the separation of ciliary muscle fibers from the scleral spur [1,2]. One of the consequences of cyclodialysis is the opening of communication between the anterior chamber and the suprachoroidal space, which forms a bypass of the aqueous humor outflow, and results in chronic ocular hypotony. It can lead to a severe decrease in visual acuity due to the corneal edema, cataract, refractive change and hypotony maculopathy [2,3,4]. Intraocular surgery involving iris manipulation, such as trabeculectomy [5], trabeculotomy, phacoemulsification [6], intraocular lens implantation [7], phakic IOL removal [8], and extracapsular cataract surgery, can give rise to iatrogenic cyclodialysis. In modern surgical practice, the formation of a surgery-associated cleft is rare. The most common causes of cyclodialysis clefts are ocular traumas, which can induce the disinsertion of the longitudinal ciliary muscle fibers from the sclera by stretching the ocular tissue due to mechanical axial compression and rapid equatorial expansion. It has been documented that injury from firecrackers, balls, stones, punches, airbags, and elastic cords could cause cyclodialysis. Meanwhile, traumatic cyclodialysis occurs as a result of blunt trauma or open globe injuries to the eye, with an incidence of 1–11% [9]. The accurate localization of the traumatic cleft is crucial for the treatment. Gonioscopy has been considered as the gold standard for cleft identification. However, clinical assessment by gonioscopy is often difficult in the soft eye with concurrent corneal edema or hyphema. In this scenario, UBM is helpful for confirming the diagnosis and gauging the size of the cleft. Traumatic cyclodialysis can coexist with anterior segment damage like iridodialysis, cataract, and lens dislocation. It is usually accompanied by chorioretinal disorders, such as retinal incarceration, hemorrhagic choroidal detachment, and choroidal avulsion, which makes it a challenge to repair cyclodialysis as well as chorioretinal injuries.

The spontaneous closure of cyclodialysis clefts is rare. Hence, various approaches for the management of cyclodialysis have been reported. Topical medical treatment (atropine) is an effective treatment for patients with small clefts. It can close the fistula through the relaxation of the ciliary muscle, which facilitates the apposition of the detached ciliary body to the sclera [1]. If medical treatment is unsuccessful, cryocoagulation or laser photocoagulation, such as argon laser, transscleral diode photocoagulation or YAG laser, are proposed to be used for closing up the clefts. These noninvasive approaches can produce local inflammatory responses in the cleft to assist apposition [2]. However, it has been demonstrated that these maneuvers have unpredictable outcomes [10]. Surgical management is the option for small clefts when the aforementioned approaches have proven ineffective, and for large cyclodialysis clefts. Cyclotamponade facilitates cleft closure by providing a mechanical tamponade effect to the detached ciliary body. Exocyclotamponade, based on the principles of retinal detachment surgery, achieves the closure of clefts by anterior scleral buckling with a short sponge or silicone tube. However, it is advised that the implant is removed after the cyclodialysis clefts are closed due to postoperative foreign-body sensations and poor cosmesis. The endocyclotamponade can exert pressure to reattach the ciliary muscle by a sulcus-inserted intraocular lens and capsular tension ring (CTR). Potential risks for this procedure include ciliary body damage, erosion, and pain from the compressive effect of the haptics or the CTR. Cyclopexy, which involves suturing the detached ciliary body to the sclera, was developed for repairing the cyclodialysis. Direct cyclopexy refers to fixing the detached ciliary body under direct visualization after creating a scleral flap and full-thickness scleral incision. The success rate of direct cyclopexy varies from 50% to 94% [11]. Internal cyclopexy, such as the sewing-machine technique and double-armed suture technique, is another surgical technique to close the cleft by an inside-out manner to avoid cutting down the sclera [12,13].The less invasive and more efficient the internal cyclopexy technique is, the better the surgical outcomes will be.

In the present study, the single 10-0 polypropylene string was used to repair cyclodialysis clefts combined with vitreoretinal injury due to blunt trauma or open-globe injury, under the guidance of a 29 G gauge needle, and the surgical outcomes of this modified internal cyclopexy technique were described.

## 2. Subjects and Methods

### 2.1. Study Design and Participants

This study was a retrospective study conducted following the principles of the Declaration of Helsinki and was approved by the Human Research and Ethics Committee of Xiangya Hospital of Central South University. Patients in this study were treated at the Department of Ophthalmology at Xiangya Hospital of Central South University from August 2020 to June 2022.

The study included patients who were diagnosed with a cyclodialysis cleft due to severe ocular trauma with one or more additional anterior or posterior segment damage indications (including cataract, lens dislocation, vitreous hemorrhage, retinal detachment, subretinal hemorrhage, choroidal detachment, and suprachoroidal hemorrhage or choroidal avulsion). Patients without regular follow-ups for at least 6 months were excluded.

### 2.2. Clinical Evaluation

Patients’ information (preoperative) including gender, age, ocular history, best-corrected visual acuity (BCVA), intraocular pressure(IOP), fundus photography, B-scan ultrasonography, and ultrasound biomicroscopy (UBM) were collected. BCVA was measured using the standard logarithmic vision chart (converted into logMAR for statistical analysis). Counting-fingers vision was converted to 1.87 LogMAR, hand-motions vision to 2.3 LogMAR, and light-perception vision to 2.8 LogMAR, and no-light-perception vision to 4 LogMAR. Intraocular pressure (IOP) was measured three times by a pneumotonometer (CT-800 computerized tonometer, Topcon Ltd., Tokyo, Japan), and the average value was adopted. Ultra-widefield fundus image was acquired with an Optos retinal imaging device (Optos, Dunfermline, UK). B-scan ultrasonography was performed to assess the condition of the vitreous, retina and choroid. Ultrasound biomicroscopy (UBM, Model SW-3200L; Tianjin Suowei Electronic Technology Co., Ltd., Tianjin, China) was conducted by two experienced doctors to examine the size of cyclodialysis cleft. To evaluate the efficacy of modified internal cyclopexy technique, the duration of fixing of the detached ciliary body was measured and displayed as surgical time (min/30° cleft). All patients were followed up at least 6 months postoperatively. BCVA, IOP, ciliary-body reattachment rates, and postoperative complications were assessed at each follow-up. Subsequent treatment was applied according to the clinical findings at each follow-up.

### 2.3. Surgical Procedure

**Figure 1:**
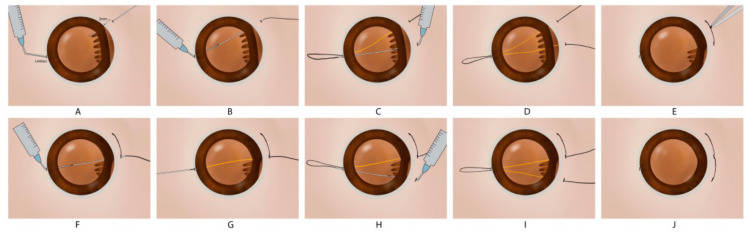
Surgical procedures of modified single-suture technique. (**A**) A needle with a 10-0 polypropylene thread was pierced into the eye at the site of the cyclodialysis cleft. (**B**) A 29-gauge needle guided the long straight needle to the outside through a corneal limbus incision. (**C**,**D**) The needle returned into the anterior chamber, and pierced the other site of the cyclodialysis cleft to the outside with the assistance of the 29-gauge needle. (**E**) The 10-0 polypropylene thread was tied onto the surface of the sclera and the suture knot was rotated into the eye. (**F**) Another suture was overlapped with the previous one by approximately 0.5 mm to avoid the residual cleft. (**G**–**J**) The suturing maneuver was repeated to attach the ciliary body to the sclera.

All surgeries were performed by Dr. Siqi Xiong at the eye center of Xiangya Hospital. Under general anesthesia or retrobulbar anesthesia, a standard 3-port 23-gauge transconjunctival pars plana vitrectomy (PPV) was performed to remove intraocular hemorrhage. The cataract or lens dislocation was removed by phacoemulsification or pars plana-based lensectomy. The conjunctiva was opened at the limbus extending 1 clock hour beyond both ends of the cyclodialysis cleft according to UBM analysis. One long straight needle with a 10-0 polypropylene thread was pierced into the eye through the sclera (2 mm posterior to the limbus) at one of the ends of the cyclodialysis cleft. A 29-gauge needle was introduced into the eye at limbus incision opposite to the cleft to guide the long straight needle passing through the ciliary body to the outside. After that, the long straight needle was returned into the eye through the same limbus incision, and the 29-gauge needle was pierced into the eye (2 mm posterior to the limbus) to guide the straight needle outside of the globe at a location about 1 clock hour distant to the first part of the thread. The 10-0 polypropylene thread was then tied to reattach the ciliary body. The knots of the suture were rotated into the sclera. The abovementioned steps were repeated until the cleft was totally repaired (Figure 1). For patients with choroidal avulsion, uveal tissues incarcerated into the wound were carefully separated by forceps and hyaluronic acid was injected into space between the “kissing choroid” to reattach the ciliary body and choroid to the inner surface of the sclera (Supplementary Video S1). For a patient with choroidal detachment or suprachoroidal hemorrhage, a balanced salt solution was intravitreally injected to increase intraocular pressure, and fluid or blood in the suprachoroidal space was drained out through sclerotomy. For a patient with traumatic iridodialysis, a 10-0 polypropylene single-arm suture technique was used to repair iridodialysis. Subsequently, the residual posterior vitreous cortex and epiretinal membrane were removed, and the internal limiting membrane (ILM) was peeled off following the injection of approximately 0.2 mL of 0.25% indocyanine green for 30 s in patients with a macular hole. Retinectomy was performed for the reattachment of incarcerated retinas. Perfluorooctane or fluid-air exchange were used to flatten the retina. The retinal breaks or retinectomy edge was treated with retinal photocoagulation. Following fluid-air exchange, C3F8 gas (14%) or silicone oil were injected into the vitreous cavity, while sclerotomies and conjunctiva were finally closed with a 8-0 polyglactin 910 suture (ETHICON, LLC. EMW9560).

Patients with a retinal detachment were asked to remain in a face-down position for at least 12 h per day for two weeks to one month. Antibiotics and corticosteroids were used topically to clear microbial infections and reduce inflammation after surgery. Silicone-oil-removal surgery and intraocular lens implantation were usually performed after three months.

### 2.4. Statistical Analyses

Statistical analyses were performed with SPSS (version 19.0, Chicago, IL, USA). Descriptive statistics included the mean and standard deviation. Preoperative and postoperative data were compared using a Wilcoxon signed-rank test. *p* < 0.05 was considered the threshold of statistical significance.

## 3. Results

A modified single-arm suture technique was performed in 20 eyes (20 men; mean age, 51.1 ± 16.2 years, ranging from 5 to 67 years). All patients were followed up for at least 6 months (range, 6 to 28 months). They included 16 eyes with an open globe injury (80%) and 4 eyes with blunt trauma (20%). zone 3 (extends more than 5 mm posterior to the limbus) was the most affected zone in open globe injuries (13 eyes, 65%), followed by zone 2 (extends posteriorly from the limbus up to 5 mm posterior to the limbus) in 2 eyes (10%) and zone 1 (cornea and limbus) in 1 eye (5%). The ruptured globe was repaired within 48 h and secondary vitrectomy was performed in an average of 15.9 ± 18.3 days (range, 5 to 90 days). Demographic and preoperative information, including age, gender, types of injury, traumatic zone and surgery time interval is shown in Table 1.

Besides cyclodialysis, all eyes had additional trauma complications including corneal wound/opacity (15%), iris injury (60%), hyphema (60%), lens extrusion (30%), lens dislocation (45%), vitreous hemorrhage (100.0%), retinal detachment (75%), macular holes (10%), choroid avulsion (5%) and suprachoroidal hemorrhage (35%). All the patients underwent 23-gauge PPV, and additional treatment procedures, such as lensectomy, iridodialysis repair, retinectomy, internal membrane peeling and intraocular tamponade, were applied accordingly, as shown in Table 2.

**Figure 2:**
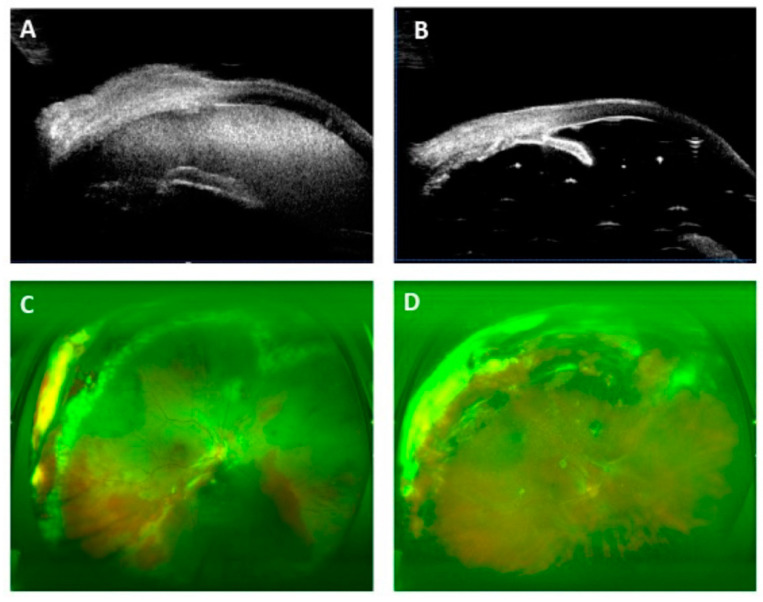
Modified single-suture technique was performed on the patient with traumatic cyclodialysis due to zone-3 ruptured-globe injury. (**A**) Preoperative UBM analysis indicated traumatic cyclodialysis combined with hyphema (white arrow). (**B**) Postoperative UBM analysis demonstrated cyclodialysis cleft was closed 3 months after silicone-oil removal. (**C**) On the first postoperative day, the retina attached with silicone oil tamponed. Retinectomy was performed from 8-1 clock in order to separate incarcerated retina from the scleral rupture. (**D**) Retina remained attached 3 months after silicone-oil extraction with LogMAR BCVA 1.0 and 14 mmHg for IOP.

**Figure 3:**
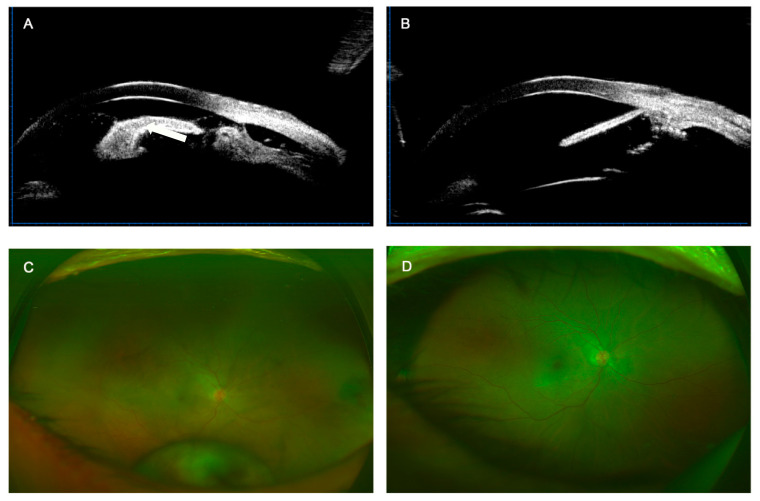
Modified single-suture technique was performed on the patient with cyclodialysis due to blunt trauma. (**A**) Traumatic cyclodialysis (white arrow) was found according to preoperative UBM analysis. (**B**) Cyclodialysis cleft was repaired 4 months after surgery. (**C**) Preoperative fundus photography indicated dislocation of lens. (**D**) Postoperative fundus photography at 2 months.

**Figure 4:**
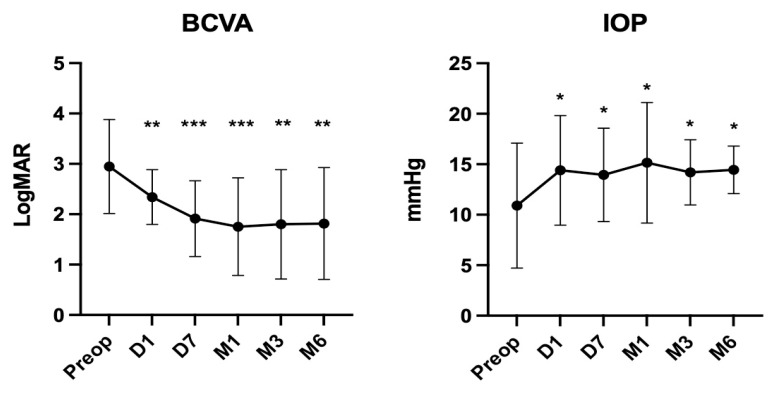
BCVA significantly improved at day 1, day 7, month 1, month 3, and month 6 compared with preoperative BCVA (*n* = 20). IOP significantly increased at day 1, day 7, month 1, month 3, and month 6 compared with preoperative IOP (*n* = 20). Abbreviation: Preop: pre-operation; D1: 1 day after cyclopexy; D7: 1 week after cyclopexy; M1: 1 month after cyclopexy; M3: 3 months after cyclopexy; M6: 6 months after cyclopexy. (* *p* < 0.05, ** *p* < 0.01, *** *p* < 0.001).

The extent of the cyclodialysis cleft was from 30° to 360°. It was larger than or equal to 180° in 11 eyes (55%) which included 1 eye with a 360° cleft (5%). The average surgical time for fixing a cyclodialysis cleft was 2.68 ± 0.54 min/30° cleft. The cyclodialysis cleft was closed in all 20 patients (100.0%) as confirmed by UBM after surgery (Figure 2 and Figure 3). Figure 4 shows the preoperative and postoperative BCVA. Compared with the preoperative BCVA (2.94 ± 0.93), postoperative BCVA showed significant improvements at day 1 (BCVA = 2.34 ± 0.54, *p* < 0.05), day 7 (BCVA = 1.91 ± 0.75, *p* < 0.05), 1 month (BCVA = 1.75 ± 0.96, *p* < 0.05), 3 months (BCVA = 1.80 ± 1.08, *p* < 0.05), and 6 months (BCVA = 1.81 ± 1.11, *p* < 0.05) postoperatively. The mean values of IOP during the follow-up period are presented in Figure 4. Compared with the preoperative IOP (10.90 ± 6.18 mmHg), postoperative IOP, including at day 1 (14.40 ± 5.42 mmHg), day 7 (13.95 ± 4.62 mmHg), 1 month (15.15 ± 5.96 mmHg), 3 months (14.20 ± 3.22 mmHg), and 6 months (14.45 ± 2.35 mmHg), was significantly increased (*p* < 0.05).

Postoperative complications included a temporary IOP spike (*n* = 4, 20%), choroidal neovascularization (*n* = 1, 5%), and silicone-oil dependency (*n* = 4, 20%). The temporary IOP spike was controlled with the topical application of antiglaucoma medication. The intravitreal injection of anti-VEGF medication was carried out to control choroidal neovascularization.

## 4. Discussion

In the current study, the modified internal cyclopexy technique was performed in 20 patients with a cyclodialysis cleft. A proportion of 55% of them were associated with a cleft of more than 180°. All patients achieved anatomical cleft closure in the first surgery. Each suture was overlapped with the previous one by approximately 0.5 mm to avoid postoperative hypotony caused by the residual cleft. In addition, these patients had combined retinal and choroid disorders, so the gas and silicone oil were used for the tamponade which could mechanically hold the detached ciliary body and choroid to the sclera by its surface tension and buoyant force. To evaluate the efficiency of this modified technique, surgical duration was measured. We found that this modified surgical technique could efficiently and accurately repair the cleft with 2.68 ± 0.54 min/30° cleft. This may be attributed to the following reasons. First, it is a flapless procedure which avoids creating a partial-thickness scleral flap and full-thickness scleral incision over the cleft, especially for large cyclodialysis clefts. It could also prevent unnecessary damage to the sclera tissue. Second, the 29 G gauge needle used in this study matched the 10-0 polypropylene string needle well, facilitating the string accurately passing through the detached ciliary body, sclera and limbus incision. Compared to the sewing-machine technique which fixes cyclodialysis by repeatedly passing the needle with the suture through the ciliary body and the sclera from the inside out, the location of the suture could be well controlled under the guidance of the 29 G gauge needle, which might increase the anatomical precision of ciliary-body reattachment. Third, the single string is enough to repair a large cleft, even in cases with the cleft extended to 360°. If a double-arm suture technique, which uses two long straight needles of a double-arm 10-0 polypropylene thread, were applied in the case of the 360° cyclodialysis cleft, the surgeon would have spent more time repeatedly knotting the two strings in order to maintain the double-arm suture. When applying this technique to a large cleft closure, it is advisable to use forceps to stabilize the detached ciliary body when passing string through it. This manipulation can avoid intraoperatively exacerbating cyclodialysis. Meanwhile, the suture knots could be easily rotated into the eye intraoperatively, which could decrease the possibility of postoperative suture erosion.

In this study, one patient experienced choroidal avulsion, whose choroid and ciliary body detached from the scleral spur and incarcerated into the sclera wound. This may be associated with a massive suprachoroidal hemorrhage, and the mechanical force destroying the adhesion of the choroid and ciliary body to the sclera [14]. It leads to a decreased IOP and eventually phthisis bulbi due to the failure in the production and excessive bypass outflow of the aqueous humor [15]. Different techniques have been developed to improve the surgical outcomes of choroidal avulsion. Medical fibrin glue, used as a topical biological adhesive, has been introduced to fix an avulsed choroid [16]. In this procedure, fibrin glue was injected into the space between the detached choroid and the sclera after fluid–air exchange, promoting the anatomical reattachment of the choroid. However, for cases of large choroidal avulsion combined with retinal incarceration, it is important to repair the damaged choroid before manipulating the detached retina, as fibrin-clot formation only occurs in a non-water surgical environment. Therefore, it becomes challenging to adhere the avulsed choroid to the sclera by fibrin glue in such complicated conditions. The incarceration of the avulsed choroid into the scleral incision has been documented. However, this technique may cause the exposure of the uvea and increase the risk of sympathetic ophthalmia postoperatively [17]. In this study, incarcerated uveal tissues were carefully separated from a scleral wound and hyaluronic acid was used to reattach the ciliary body and choroid to the inner surface of the sclera, which could decrease the possibility of damaging the avulsed choroid while manipulating the fragile choroidal tissue. We found that the choroid and ciliary body were anatomically attached to the sclera with this modified single-arm suture technique, indicating that it is suitable for choroidal avulsion with ciliary body detachment as a whole unit. Further investigations with a larger sample size and a longer follow-up period are required to figure out the therapeutic effect of this modified single-armed suture technique on choroidal avulsion.

Cyclodialysis occurred in four patients due to blunt trauma, which was also combined with lens dislocation, iridodialysis, vitreous hemorrhage and retinal detachment. In patients with open globe injury, cyclodialysis often concurred with incarcerated retinal tissues, hemorrhagic choroidal detachment and choroidal avulsion. Therefore, the surgical outcomes of patients with cyclodialysis caused by blunt trauma were more favorable than those of patients with open globe injury. However, the cyclodialysis cleft was anatomically repaired, and the blood clot and exudative membrane were completely removed intraoperatively to prevent the formation of ciliary body membranes, which can damage the ciliary processes and lead to a cessation of aqueous production. Four patients (20%) with open globe injury involving zone 3 were silicone-oil-dependent (patients required permanent silicone-oil tamponade to prevent persistent hypotony and phthisis bulbi). The dysfunction of the ciliary body due to severe injury, and the exposure of the bare retinal pigment epithelium associated with large retinectomy for the reattachment of incarcerated retinal tissues, were the main reasons for silicone-oil dependence in these eyes.

It has been reported that a transient IOP spike is common in patients with cyclodialysis cleft repair [18]. The mechanisms underlying the IOP increase after the successful closure of the cleft remain unclear. One hypothesis suggests that the increased uveoscleral outflow through the cleft may promote the production of aqueous humor by the ciliary body, and disrupt the drainage function of the trabecular meshwork. Elevated IOP can occur when these compensatory changes in aqueous production and trabecular outflow take time to normalize after cleft closure [19]. The incidence of an IOP spike in the early postoperative period varies depending on the treatment modalities adopted. In this study, we found that 20% of patients experienced a transient IOP increase, which was controlled by the local administration of ocular hypotensive eyedrops. None of our patients developed a persisting elevated IOP or glaucoma. Choroidal neovascularization (CNV) was found in 5–12% of cases of ocular trauma with choroidal rupture. It is associated with the disruption of RPE, the Bruchs membrane, and choriocapillaris [20]. One patient developed choroidal neovascularization postoperatively, which was successfully controlled by anti-VEGF treatment. No other serious postoperative complications, such as hemorrhage from the ciliary body or endophthalmitis, occurred in this study, suggesting that this modified cyclopexy is safe for ameliorating cyclodialysis clefts due to severe ocular trauma.

As the sample size of this study was small, it was difficult to compare the surgical outcomes between cases with blunt trauma and open globe injury statistically. Further research with larger groups and a long-term follow-up is required to detail the efficacy and complications of this modified technique.

## 5. Conclusions

A modified single-suture technique could efficiently close cyclodialysis clefts and facilitate the repair of intraocular disorders in patients with blunt trauma or open globe injury.

## Figures and Tables

**Table 1:** Basic characteristics of enrolled patients.

Objectives	Patients Enrolled (*n* = 20)
Age, mean ± SD	51.1 ± 16.2
Gender, *n* (%)	
Male	20 (100%)
Female	0 (0%)
Type of injury, n (%)	
Open globe Injury	16 (80%)
Blunt trauma	4 (20%)
Traumatic zone, *n* (%)	
I	1 (5%)
II	2 (10%)
III	13 (65%)
Surgery time interval, mean ± SD (days)	15.9 ± 18.3

SD, standard deviation; *n*, number; surgery time interval: time interval between the occurrence of trauma and cyclopexy.

**Table 2:** Ocular characteristics and surgical interventions.

	Patients Enrolled (*n* = 20)
Cornea wound/opacity, *n* (%)	3 (15%)
Iris injury, *n* (%)	12 (60%)
Cyclodialysis cleft, *n* (%)	
<180°	9 (45%)
≥180°, <360°	10 (50%)
360°	1 (5%)
Hyphema, *n* (%)	12 (60%)
Lens, *n* (%)	
Extrusion	6 (30%)
Dislocation	9 (45%)
Vitreous hemorrhage, *n* (%)	20 (100.0%)
Retina detachment, *n* (%)	15 (75%)
Macular hole, *n* (%)	2 (10%)
Choroid avulsion, *n* (%)	1 (5%)
Supra choroidal hemorrhage, *n* (%)	7 (35%)
Surgical interventions, *n* (%) Cyclodialysis cleft repair	20 (100%)
Lensectomy/Phacoemulsification	12 (60%)
Iridodialysis repair	5 (25%)
Retinectomy	13 (65%)
Silicone oil	15 (75%)
Surgical time for repairing cyclodialysis (min/30° cleft)	2.68 ± 0.54

*n*, number.

## Data Availability

The data underlying this article are available in the article.

## Supplementary Materials

The following supporting information can be downloaded at: https://www.mdpi.com/article/10.3390/jcm12134252/s1, Video S1: Repair traumatic cyclodialysis cleft combined with chorioretinal injury by modified single-armed suture technique.
